# Two cases of interparietal inguinal hernias undergoing laparoscopic treatment: a case series

**DOI:** 10.1093/jscr/rjad051

**Published:** 2023-02-14

**Authors:** Hiroki Ozawa, Asuka Hara, Keita Hayashi, Yasushi Kaneko, Hiroto Kikuchi, Hiroto Fujisaki, Akira Hirata, Kumiko Hongo, Kiminori Takano, Atsushi Suga, Kikuo Yo, Kimiyasu Yoneyama, Motohito Nakagawa

**Affiliations:** Department of Surgery, Hiratsuka City Hospital, Hiratsuka City, Kanagawa, Japan; Department of Surgery, Hiratsuka City Hospital, Hiratsuka City, Kanagawa, Japan; Department of Surgery, Hiratsuka City Hospital, Hiratsuka City, Kanagawa, Japan; Department of Surgery, Hiratsuka City Hospital, Hiratsuka City, Kanagawa, Japan; Department of Surgery, Hiratsuka City Hospital, Hiratsuka City, Kanagawa, Japan; Department of Surgery, Hiratsuka City Hospital, Hiratsuka City, Kanagawa, Japan; Department of Surgery, Hiratsuka City Hospital, Hiratsuka City, Kanagawa, Japan; Department of Surgery, Hiratsuka City Hospital, Hiratsuka City, Kanagawa, Japan; Department of Surgery, Hiratsuka City Hospital, Hiratsuka City, Kanagawa, Japan; Department of Surgery, Hiratsuka City Hospital, Hiratsuka City, Kanagawa, Japan; Department of Surgery, Hiratsuka City Hospital, Hiratsuka City, Kanagawa, Japan; Department of Surgery, Hiratsuka City Hospital, Hiratsuka City, Kanagawa, Japan; Department of Surgery, Hiratsuka City Hospital, Hiratsuka City, Kanagawa, Japan

## Abstract

Interparietal inguinal hernia, an exceedingly rare type of inguinal hernia in which the hernia sac anatomically lies between the tissue layers of the abdominal wall, is difficult to diagnose from physical findings. Given the few reports on interparietal inguinal hernias, this condition has remained fairly unrecognized. Herein, we report the successful imaging and laparoscopic diagnoses as well as repair of an interparietal inguinal hernia. Atypical physical findings and computed tomography data help in the diagnosis of an interparietal inguinal hernia. The laparoscopic approach is useful and feasible for both the diagnosis and treatment of interparietal inguinal hernia.

## INTRODUCTION

Interparietal hernia is a type of hernia in which the hernia sac extends into various muscle layers and abdominal wall muscles. To the best of our knowledge, the first case of interparietal hernia was reported by Bartholin in 1661 [1]. Moreover, Lower and Hicken [1] reported an incidence rate of 0.02–1.6% after examination. However, the actual rate could be much higher than that reported in these studies as several cases remain undiagnosed. Interparietal hernias can be classified into the following three types: preperitoneal (between peritoneum and transversalis fascia), interstitial (between transversalis fascia and transverse, internal oblique or external oblique muscles) and superficial hernias (between the external oblique and skin or within aponeurosis of the inguinal region). Interstitial hernias are the most common type of interparietal hernia (accounting for ~60%), with preperitoneal and superficial hernias accounting for 20% each [1]. Men are more likely than women to develop interparietal hernias, with the average age of occurrence being 30–40 years for men and 50–60 years for women [2]. Spigelian hernias, in which the hernial orifice is located at the semilunar line and the Spigelian aponeurosis at the outer border of the rectus abdominis, have also been included in the broad characterization of interparietal hernias [3]. Given its clinical ambiguity and rare frequency, this condition can be difficult for surgeons to diagnose preoperatively.

Although the definition of interparietal inguinal hernias is ambiguous, they form a group of rather unusual hernias located between the layers of the abdominal wall in the inguinal region [4]. The etiology of interparietal inguinal hernias is considered to be both congenital and mechanical. The congenital factors include the inadequate descent of the testis, congenital depression of the peritoneum and dysplasia of the external inguinal ring. The mechanical factors include the obstruction of the inguinal canal by the canal of Nuck or the ovaries in a normal inguinal hernia [2, 5, 6].

Herein, we present two cases of patients with interparietal inguinal hernias who underwent laparoscopic treatment after diagnosis. Interparietal inguinal hernia was defined as an interparietal hernia of the inguinal region in this report.

## CASE SERIES

### Case 1

**Figure 1:**
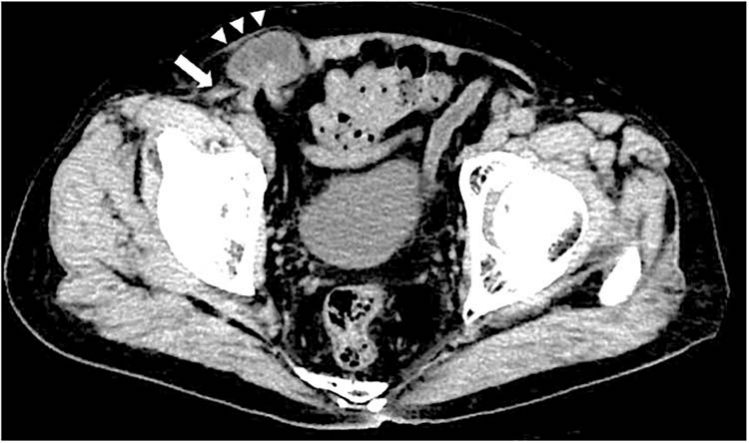
Contrast-enhanced CT scan; an incarcerated bowel was observed in the right inguinal region; the small bowel was incarcerated between internal and external oblique muscles; the white arrow indicates the internal oblique muscle, and the white triangles indicate the external oblique muscle.

**Figure 2:**
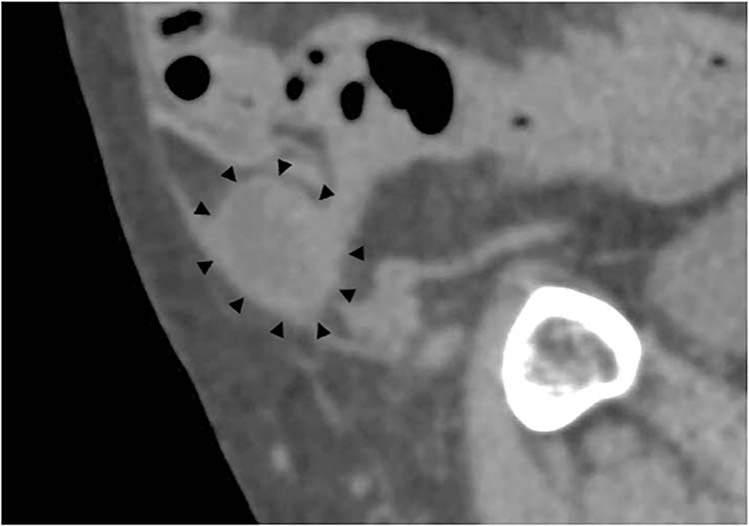
Sagittal section of contrast-enhanced CT; the black triangles indicate the incarcerated small bowel.

A 79-year-old male patient with a medical history of right inguinal hernia presented to the emergency department with right inguinal swelling and pain. We found the inguinal swelling, which extended toward the head, to be atypical. In addition, contrast-enhanced computed tomography (CT) scan revealed that a portion of the small intestine was incarcerated between the internal and external oblique muscles, and the hernia contents had prolapsed cranially from the hernial orifice. Furthermore, the sagittal view revealed that the contents of the hernia had prolapsed ventrally and not along the spermatic cord (Figs 1 and 2). Therefore, we diagnosed it as a right interparietal inguinal hernia, and laparoscopic hernia repair was planned. Owing to the fact that the manipulative reduction was difficult, we planned to release the incarceration first.

**Figure 3:**
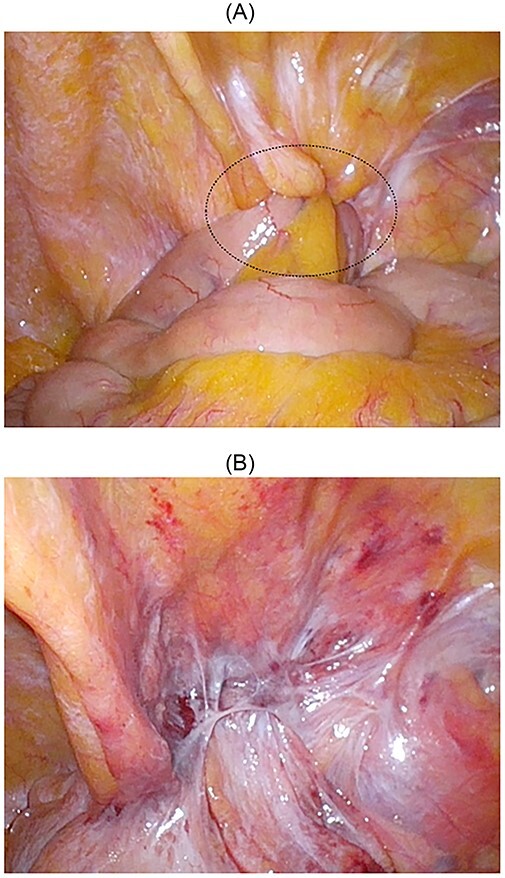
Intraoperative findings; the small bowel was incarcerated; the dotted circle indicates the peritoneal swelling by hernia contents (**A**); after manupulation, the hernial orifice could not be found (**B**).

**Figure 4:**
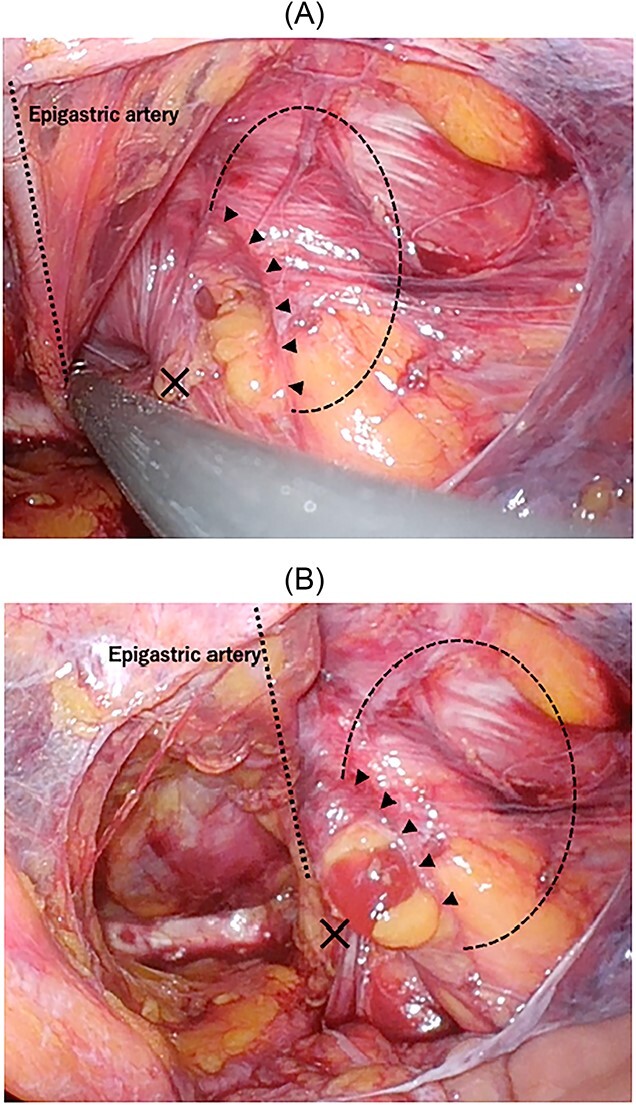
Intraoperative findings of right inguinal region after peritoneal incision; the black triangles show the hernial orifice; the dotted circle indicates the assumed hernia sac; the black cross indicates the internal inguinal ring (**A**); by pressing the inguinal region from the body surface, intermuscular soft tissue was prolapsed into the abdominal cavity (**B**).

A laparoscope was inserted through a 12-mm umbilical port. Another 12-mm port was created at the level of the umbilicus aligning with the right mid-clavicle, and a 5-mm port was placed on the contralateral side of the line connecting the hernial orifice to the umbilicus. The bulging in the abdominal cavity caused by the hernia’s content could be observed in the laparoscopic view (Fig. 3A). Laparoscopic reduction was successfully performed, and a small segment of the incarcerated bowel was mildly erythematous. Therefore, we decided that bowel resection was unnecessary. After observing the hernial orifice, we found that the peritoneum with the right medial umbilical fold was easily turned inward into the abdominal cavity, suggesting a type of sliding hernia involving the medial umbilical folds [7], and the hernial orifice could not be identified (Fig. 3B). After the peritoneal incision and dissection, the hernial orifice was found lateral to the right epigastric artery. However, although the inner inguinal ring was small, the hernial orifice was observed under the transversus aponeurotic arch in the ventral–lateral direction (Fig. 4A). By pressing the inguinal region from the body surface, the hernial orifice could be recognized more easily (Fig. 4B). The size of the hernial orifice was ~20 mm. Based on the aforementioned findings, the patient was rediagnosed with right interparietal inguinal hernia. After diagnosis, laparoscopic preperitoneal hernia repair was performed with a mesh using the same surgical procedure as that for a standard transabdominal preperitoneal (TAPP) repair. In addition, the edge of mesh was placed ≥3 cm from the hernial orifice, and the center of the mesh was slightly ventral and lateral to the internal inguinal ring.

On postoperative Day 2, the patient resumed oral intake, and was discharged on postoperative Day 4 without any complications.

### Case 2

**Figure 5:**
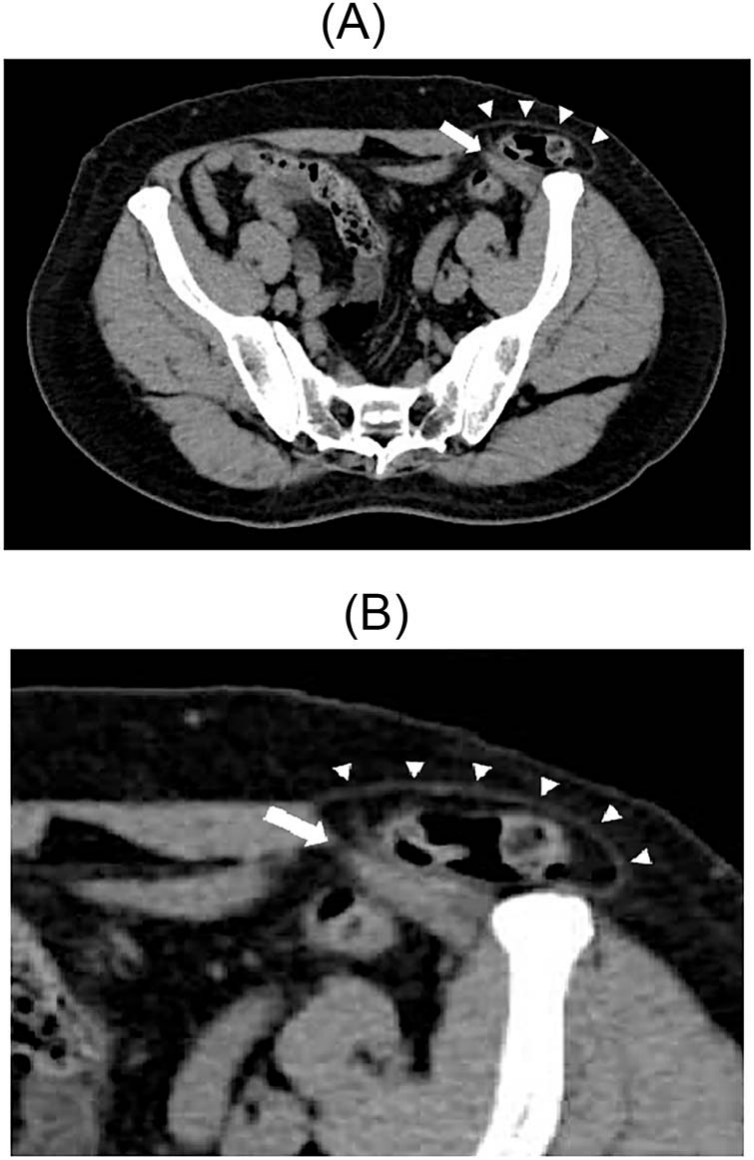
Contrast-enhanced CT scan; the sigmoid colon entered into the layer between the internal and external oblique muscles; the white arrow indicates the internal oblique muscle, and the white triangles indicate the external oblique muscle (**A**). Enlarged view of the inguinal region (**B**).

A 67-year-old male patient presented to the outpatient clinic with bilateral inguinal swelling and pain that had continued from a year prior. The swelling on the left side extended from the inguinal region toward the head. Based on the physical findings, we decided to perform CT. On the left side, contrast-enhanced CT scan revealed that a portion of the sigmoid colon protruded between internal and external oblique muscles passing near the spermatic cord. Furthermore, the sigmoid colon had prolapsed cranially from the hernial orifice. On the right side, prolapse of adipose tissue was observed (Fig. 5A and B). Therefore, the patient was diagnosed with a left interparietal inguinal hernia and right inguinal hernia. Based on this diagnosis, laparoscopic repair of both hernias was planned.

**Figure 6:**
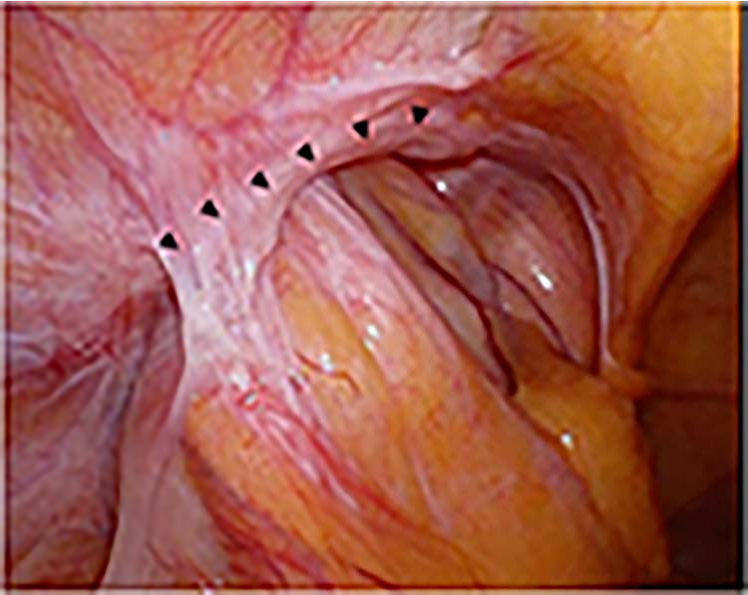
Intraoperative findings; the sigmoid colon entered into the hernial orifice as a sliding hernia; the black triangles indicate the hernial orifice.

**Figure 7:**
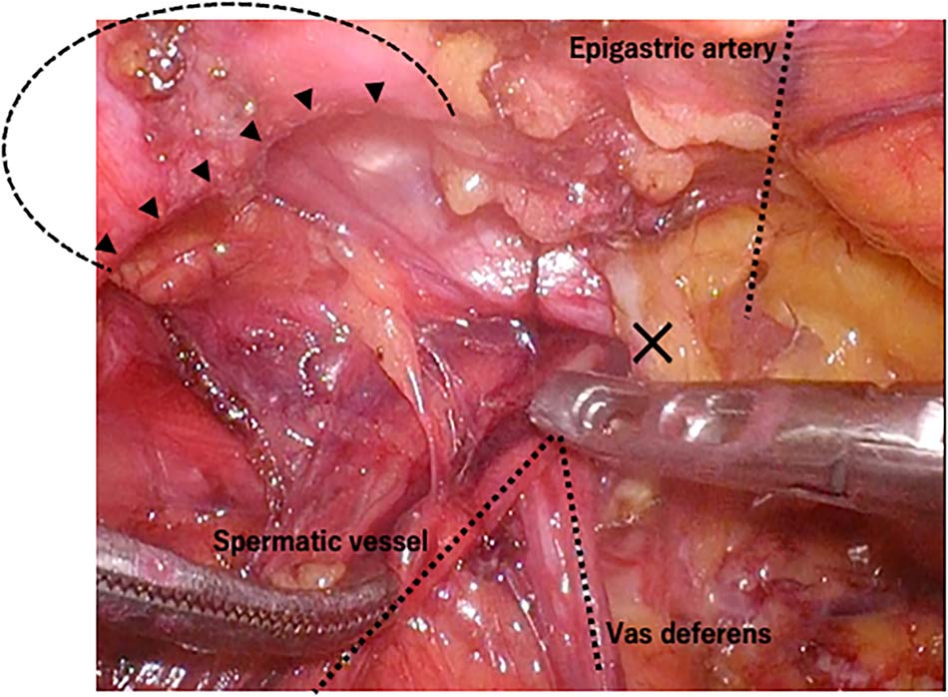
Intraoperative findings of the left inguinal region after peritoneal incision; the black triangles indicate the hernial orifice; the dotted circle indicates the assumed hernia sac; the black cross indicates the internal inguinal ring.

A laparoscope was inserted through a 12-mm umbilical port. In the laparoscopic view, on the left side, the sigmoid colon had retracted into the hernia lateral to the left epigastric artery, suggesting a sliding hernia of the sigmoid colon (Fig. 6). The size of the hernial orifice was ~30 mm. On the right side, the direct inguinal hernia was 20 mm in size. Accordingly, 12-mm ports were created at the level of the umbilicus aligning with the left and right mid-clavicle. The left side had been repaired first. After peritoneal incision and preperitoneal space dissection, the inner inguinal ring was found to be too small relative to the physical findings. Instead, the hernial orifice was observed under the conjoint tendon toward the ventral dorsal direction (Fig. 7). Based on the aforementioned findings, the left interparietal inguinal hernia was reconfirmed. Subsequently, preperitoneal repair of the hernia was performed with a mesh using the same surgical procedure as that for a standard TAPP repair. The hernia was repaired in the same way on the right and left sides. On postoperative Day 1, the patient resumed oral intake. Further, on postoperative Day 2, he was discharged uneventfully.

## DISCUSSION

An interparietal inguinal hernia is an unusual type of hernia located between the layers of the abdominal wall in the inguinal region. A laparoscopic approach is useful for diagnosing and treating interparietal inguinal hernias. Furthermore, this type of hernia can be repaired by the same procedure as that for a standard TAPP repair.

There have been only three previous reports of interparietal inguinal hernia (Table 1). Kumar *et al*. [8] reported a giant interparietal inguinal hernia with undescended testis for which they performed preperitoneal placement of a proline mesh and orchiopexy. Meanwhile, Sakamoto *et al*. [9] reported the successful laparoscopic reduction and repair of a strangulated interparietal inguinal hernia. They suspected an interparietal or internal hernia near the right groin based on the CT scan. Given the ambiguous diagnosis, laparoscopic exploration was planned. Hirabayashi *et al*. [4] reported a case with the combination of an interparietal inguinal hernia and ipsilateral ectopic testicle. Because of the failure of the gubernaculums to migrate to the scrotum, the inguinal canal space was not well developed, and the hernia sac expanded between the external oblique aponeurosis and internal oblique muscle as well as through the inguinal canal. The definitions of interparietal hernias presented in these three reports are ambiguous. Given the anatomical characteristics of the inguinal region, interparietal inguinal hernias have to be departed from other interparietal hernias. Furthermore, an interparietal inguinal hernia could be defined as the interparietal hernia near the external inguinal ring. Based on the abovementioned reasons, the number of interparietal inguinal hernias in clinical settings may have been more than the reported number.

**Table 1:** Clinical outcomes of the interparietal hernia

**Author**	**Publish year**	**Age**	**Sex**	**Side**	**Symptom**	**Preoperative diagnosis**	**Type**	**Operative procedure**
Hirabayashi [4]	2013	22 days	male	right	Swelling from the right lumbar region to the right iliac fossa region	Spigelian hernia Ectopic Testis	interstitial	Laparoscopic examination Anterior approach (High ligation and orchiopexy)
Sakamoto [9]	2016	51 years	male	right	Pain (no swelling)	Strangulated inguinal hernia or interparietal hernia	preperitoneal	TAPP
Kumar [8]	2018	62 years	male	right	Huge swelling over the right lower abdomen	Spigelian hernia	interstitial	Anterior approach (preperitoneal placement of proline mesh and orchiopexy)
Our case1	2022	79 years	male	right	Swelling and pain	strangulated inguinal hernia	interstitial	TAPP
Our case2	2022	67 years	male	left	Swelling from the inguinal region toward the head	interparietal hernia	interstitial	TAPP

TAPP: transabdominal preperitoneal repair

Considering the difficulty in establishing a diagnosis preoperatively, the diagnosis of interparietal inguinal hernia is usually made intraoperatively. The expansion of the inguinal swelling is usually palpable toward the head, as seen in case 2 [10–12]. This physical finding may be helpful for the preoperative diagnosis of interparietal inguinal hernias. If an atypical hernia protrusion is identified, imaging tests, such as CT and ultrasound, should be planned; however, imaging studies are not routinely performed for inguinal hernias. In particular, CT is noninvasive, takes less time and can provide us with a wealth of anatomical information. Therefore, it is strongly recommended. Moreover, it can help diagnose an interparietal inguinal hernia when the pathobiology is known. Specifically, the hernia contents extend into various muscle layers and abdominal wall muscles. The prolapse of hernia contents cranially to the hernial orifice is also a feature seen on CT scan. In addition, sagittal CT scans make the identification of atypical hernias simple. As mentioned earlier, a history of undescended testis or inguinal surgery may also be useful for diagnosis.

Laparoscopic approach offers various advantages. First is the visibility of the hernial orifice. In particular, the hernial orifice of interparietal hernia is located at atypical sites, such as between the muscles, rather than the internal inguinal ring. Hence, the hernial orifice, which is difficult to detect using the anterior approach, can be observed and diagnosed using the laparoscopic approach. Second, the ability to detect the hernial orifice enables hernia repair, even in cases where the diagnosis is not confirmed. Interparietal hernias have been known for a long time; however, they may be recognized again using the laparoscopic surgery. Third, considering that the preperitoneal hernia usually involves two sites, the anterior approach may only be able to detect the site extending down into the inguinal canal but not the site that passes into the interstitial space [1]. However, the TAPP can provide an internal view of the abdominal cavity. Therefore, it can completely repair the hernia. If the anterior approach reveals that the sac attached to the spermatic cord is smaller than that found on physical examination, an interparietal hernia should be suspected. Fourth, the laparoscopic approach is useful for confirming the condition of the strangulated intestinal tract after manipulative reduction.

The repair of interparietal inguinal hernias is performed using the same laparoscopic surgical procedure that is used for a standard TAPP. Given the proximity of the hernial orifice and inner inguinal ring, the interparietal hernia can be repaired by placing a mesh at the myopectineal orifice, which is an area involving the inguinal ring and hernial orifice. Moreover, the additional procedures are not strictly necessary with TAPP repair. The mesh would be placed with a margin of 3 cm to ensure that it corresponds to the hernial orifice, which is slightly displaced from the internal inguinal ring. At our institution, the ratio of TAPP to the anterior approach for inguinal hernias is 1:1, and the procedure is chosen based on the case. Therefore, TAPP is our preferred choice of treatment for an interparietal hernia.

Regarding the disadvantages, TAPP is not suitable for patients with a history of abdominal surgery, especially urologic surgery, owing to adhesions. Moreover, the medical costs are higher for TAPP.

CT is recommended for inguinal hernias with atypical physical findings; moreover, an interparietal inguinal hernia can be diagnosed preoperatively. We found that the laparoscopic approach for interparietal inguinal hernia is useful for the diagnosis and treatment. Moreover, it can be repaired using the same procedure as that for a standard TAPP.

## DECLARATIONS

This manuscript has not been published elsewhere. All the authors contributed significantly to the work and approved the final version of the manuscript.

## CONSENT FOR PUBLICATION

Consent to publish was obtained from the patients.

## DATA AVAILABILITY

All data generated or analyzed during this study are included in this published article.

## CONFLICT OF INTEREST STATEMENT

The authors declare that they have no competing interest.

## FUNDING

We have no source of funding for this article.

## AUTHORS’ CONTRIBUTIONS

H.O. is the first author and prepared the manuscript. All authors have read and approved the final manuscript.
